# Microcurrent Stimulation at Shenmen Acupoint Facilitates EEG Associated with Sleepiness and Positive Mood: A Randomized Controlled Electrophysiological Study

**DOI:** 10.1155/2015/182837

**Published:** 2015-02-12

**Authors:** Mei-chun Cheung, Agnes S. Chan, Joanne Yip

**Affiliations:** ^1^Department of Social Work, The Chinese University of Hong Kong, New Territories, Hong Kong; ^2^Department of Psychology, The Chinese University of Hong Kong, New Territories, Hong Kong; ^3^Chanwuyi Research Centre for Neuropsychological Well-Being, The Chinese University of Hong Kong, New Territories, Hong Kong; ^4^Institute of Textiles and Clothing, The Hong Kong Polytechnic University, Hung Hom, Hong Kong

## Abstract

To examine the electrophysiological effects of microcurrent stimulation at the Shenmen acupoint, 40 healthy normal subjects were randomly assigned to a placebo group (sham stimulation) and an experimental group (bilateral electrocutaneous stimulation at the Shenmen). The following two electroencephalographic indicators were used to measure brain activity. (1) Arousal level was measured with reference to log-transformed absolute alpha power and power source and analyzed using low-resolution electromagnetic tomography and (2) frontal alpha asymmetry was used as an indicator of mood. After real stimulation for 10 minutes, absolute alpha power was globally reduced in the experimental group, particularly in the anterior and centrotemporal regions of the brain. This indicates a decline in the brain activity associated with arousal. Moreover, the reduction was more prominent in the left frontal region, as compared to the right frontal region, resulting in significant increase from negative to positive frontal alpha asymmetry scores and reflecting an increase in the brain activity associated with enhanced mood. However, the placebo group exhibited no significant changes in two indicators after sham stimulation. This study provides initial electrophysiological evidence of changes in brain activity associated with reduced arousal (and thus greater sleepiness) and enhanced mood after microcurrent stimulation at the Shenmen acupoint.

## 1. Introduction

Regarded as a novel and alternative treatment modality, microcurrent stimulation has been used for decades to treat various physical and psychological problems [1]. Applying a low-intensity, direct current that delivers monophasic or biphasic pulsed microamperage currents across the brain has been found to reduce anxiety, depression, and sleep problems [2–7] and improve cognitive function [8–13]. In addition, the therapeutic effects of microcurrent stimulation on pain management [14–22] and muscle [23] and wound [24, 25] healing have been demonstrated. Recently, microcurrent stimulation has been shown to relieve the side effects of radiation therapy [26] and myocontracture in children with cerebral palsy [27]. It is also used as an adjunct treatment for patients with fibromyalgia [28–30] and in the management of diabetes mellitus and hypertension, which are well controlled after several months' stimulation [31, 32].

However, despite ample evidence of the therapeutic effects of microcurrent stimulation, its mechanism of action remains unclear. Some researchers have speculated that microcurrent stimulation affects energy that has an interactive and regulatory function in the body's communication systems [33]. Therefore, this kind of stimulation may be an effective treatment for various physical and psychological problems. Although more empirical investigation is necessary to identify its underlying mechanism of action, the benefits of microcurrent stimulation have been commonly observed in traditional Chinese medicine (TCM), and the principles of TCM may shed some light on this issue. According to TCM practitioners, our health and well-being are closely related to our internal energy and physical and psychological problems are due to imbalances in internal energy or blockages of energy flow. An interconnecting network of numerous acupoints is located on the 14 main meridians of the human body [34], and each acupoint has a defined therapeutic function [35]. Stimulation of these acupoints, whether administered by acupuncture [36], acupressure [37, 38], or an electrical device [39–41], can balance or unblock internal energy. Therefore, such stimulation has been found to correlate with various therapeutic effects [42, 43], such as pain reduction [44–46], alleviation of headaches [47–49], and management of sleep disturbance [50, 51]. Recently, researchers have also sought to determine the efficacy of acupoint stimulation in clinical populations. It appears to be effective in treating various kinds of physical and psychological problems, such as back pain [52–54], chronic pain [55–57], asthma [58–60], stroke [61, 62], insomnia [63–65], anxiety disorders [66], and smoking addiction [67–69]. If microcurrent stimulation is capable of restoring an unimpeded flow of energy through the body, as conceptualized by TCM practitioners, more insight can be gained into its observed therapeutic effects on a variety of physical and psychological problems.

With the advent of neuroimaging techniques such as functional magnetic resonance imaging and EEG, it is possible to explore the neural or electrophysiological mechanisms of TCM and internal energy in a more scientific manner [70–76]. TCM practitioners have emphasized the role of the Shenmen acupoint, located at the wrist crease on the radial side of the flexor carpi ulnaris tendon, between the ulna and the pisiform bones, in reducing sleep disturbance [64, 77, 78] and improving mood [77, 78]. Consequently, this acupoint has received particular attention in recent neuroimaging studies [79] and clinical studies [37, 38, 80–82]. Stimulation administered to the Shenmen acupoint by acupuncture results in functional activation in various brain regions [79], such as the right postcentral gyrus (BA1 and BA2), the left postcentral gyrus (BA43), the left inferior frontal gyrus (BA47), the left superior temporal gyrus (BA22), and the right inferior parietal gyrus (BA40). Administering magnetic stimulation to the Shenmen acupoint makes EEG signals less chaotic, suggesting that the brain is calmer and more ordered as a result of this treatment [83]. Positive results such as improved sleep quality [37], better quality of life [38], reduced insomnia in stroke patients [81, 82], and the reduction of psychological stress [37] have been reported in clinical trials. If microcurrent stimulation has a similar mechanism of action by restoring the flow of internal energy, the use of microcurrent to stimulate the Shenmen acupoint is also expected to increase sleepiness and enhance mood.

To investigate the effects of microcurrent stimulation at the Shenmen acupoint, two electrophysiological indices, namely, absolute alpha power and frontal alpha asymmetry, were used in this study to examine changes in brain activity associated with sleepiness and mood, respectively. Sleep research indicates that insomnia is highly associated with abnormal brain activity. Patients with insomnia have been found to exhibit hyperarousal [84] or greater global brain metabolism while awake, as compared to sleep state [85]. In particular, their resting EEG activity in both the alpha band and the nonalpha band is higher than that of normal subjects, and there is a significant positive correlation between hyperarousal scores and alpha activity on the left side when patients' eyes are open [86]. Therefore, increased EEG alpha activity seems to be associated with hyperarousal in patients with insomnia. The results of previous EEG studies also indicate that subjective sleepiness negatively correlates with absolute alpha power at all scalp locations when awake [87, 88] and that a reduction in alpha power may reflect a reduction of activation in the subcortical brain structures with general cortical activation properties, such as brain stem, midbrain, hypothalamus, and other parts of the limbic system [88]. It is therefore speculated that microcurrent stimulation at the Shenmen acupoint helps reduce absolute alpha power, thereby lowering the individual's arousal level and encouraging sleepiness.

A number of EEG studies [89–100] have investigated the relationship between EEG signals and mood and reported a link between asymmetry in the alpha-frequency band and mood. The results of these EEG studies have suggested that different emotions are associated with different EEG patterns in the frontal regions of the brain. Specifically, alpha asymmetry in the anterior frontal region between the two hemispheres of the brain is regarded as an effective index of positive or negative emotion. Positive emotions such as happiness are associated with relatively greater left-sided activation [95, 97, 99, 101], and negative emotions such as disgust are accompanied by relatively greater right-sided activation [96, 98]. As alpha power is inversely associated with brain activation in the frontal cortical region [102, 103], a positive asymmetry score denoting greater alpha power on the right and less alpha power on the left suggests relatively greater left-sided activation, that is, a more positive emotional response. In contrast, a negative score denoting greater alpha power on the left and less alpha power on the right represents relatively greater activation on the right side, suggesting a more negative emotional response. Our empirical and clinical studies [90–92] have shown that frontal alpha asymmetry provides an effective and reliable means of distinguishing between positive and negative emotions. Greater left-sided activation is also associated with reduced anxiety and feelings of well-being [90–94, 98, 100]. Therefore, if microcurrent stimulation at the Shenmen acupoint leads to an improvement in mood, a change in brain activity measured by frontal alpha asymmetry may be observed after stimulation and the reduction in absolute alpha power should be more prominent in the left frontal region, as compared to the frontal region in the right hemisphere, resulting in a positive frontal alpha asymmetry score after stimulation.

## 2. Subjects and Methods

### 2.1. Participants

Forty university students from the Institute of Textiles and Clothing at The Hong Kong Polytechnic University were recruited to the study and randomly assigned into the placebo (*N* = 20, age: 20.90 ± 1.17; years of education: 15.05 ± 0.22, GPA: 3.02 ± 0.33) and experimental (*N* = 20, age: 20.63 ± 0.83; years of education: 15.11 ± 0.32, GPA: 3.20 ± 0.43) groups. Two groups were matched in terms of age, education, and GPA. They reported a negative history of neurological and psychiatric problems. The study was conducted in accordance with the Helsinki Declaration of the World Medical Association Assembly, and the research protocol was approved by the Human Subjects Ethics Subcommittee (HSESC) of The Hong Kong Polytechnic University. All of the students participated voluntarily and were required to sign informed-consent forms prior to the study, in accordance with institutional guidelines.

### 2.2. EEG Recording

The EEG was made using 64 Ag/AgCl-sintered electrodes mounted in a stretch-lycra Quik-Cap (Neuroscan, El Paso, TX, USA) with electrode placement in accordance with the international 10-10 system [104–106]. A ground electrode was placed on the forehead of each participant anterior to Fz. The standard reference electrode of the cap, placed between Cz and CPz, was used during acquisition. Measurements of vertical electrooculography (VEOG) were taken between electrodes placed on the supraorbital and suborbital regions of the left eye, and measurement of horizontal electrooculography (HEOG) was taken between electrodes placed on the outer canthi of the left and right eyes. The impedance of the electrode was less than 10 kΩ and homologous sites were within 1 kΩ of each other. Quik-Gel (El Paso, TX, USA) was used as the conducting medium. The signals were amplified with a Neuroscan SynAmps^2^ amplifier unit (EL Paso, TX, USA) with a bandpass of 0.05 to 200 Hz and digitized at a sampling rate of 1000 Hz.

### 2.3. Experimental Procedure

**Figure 1:**
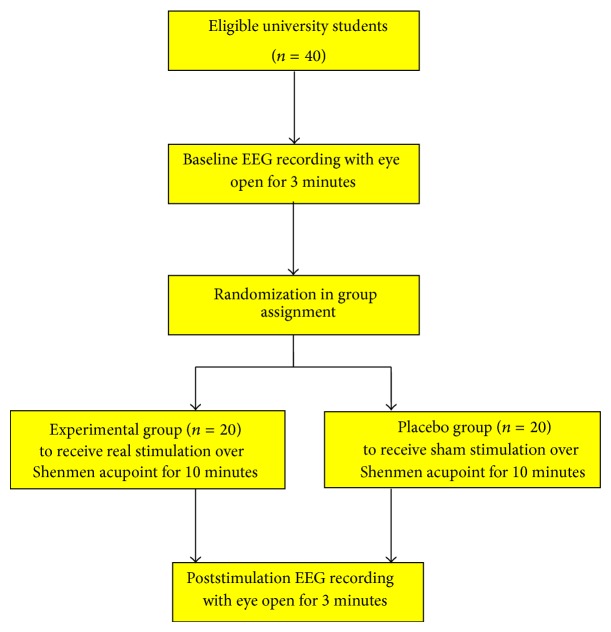
Diagram of study design.

All of the participants were tested individually in a sound- and light-isolated room. The experimental procedure was explained to each participant before the experiment began. The design of the study is shown in Figure 1. Measurements were obtained for a 3-minute baseline period during which the participants rested while awake with their eyes open. The members of the experimental group received a 10-minute noninvasive stimulation administered by a preprogrammed pulse generator. This device produced constant current square wave electrical stimulation. A symmetrical monophasic square wave with a frequency of 20 kHz was modulated by a 1500 Hz symmetrical monophasic square wave, yielding a 20 kHz pulse signal that was only activated at a 50% duty cycle (0.3 ms) during the full cycle of the 1500 Hz wave. The polarity of this signal was later reversed periodically at a frequency of 100 Hz, with no painful stimulation applied to the skin at the Shenmen acupoint (HT7) after the baseline EEG measurement. EEG activity was recorded for another 3 minutes after the 10-minute stimulation, with the participants' eyes open. The same procedure (baseline EEG measurements, stimulation, and poststimulation EEG measurements) was used with the placebo group, except that its members received a sham stimulation, as the electrical stimulation was turned off during the second phase. Sham stimulation and real stimulation were randomly administered, and the participants were blind to the group assignment; that is, they did not know which type of stimulation they had received.

### 2.4. EEG Analysis

Artifacts were removed from the EEG data during offline processing, and the data were remontaged to create a linked-ears reference scheme, using the NeuroGuide software program (NeuroGuide, v.2.5.2). Split-half reliability tests and test-retest reliability tests were used to examine the selected EEG segments. Only segments with at least 1 minute of artifact-free data and >90% reliability were used in the subsequent spectral analysis. Fast Fourier transformation was used to translate the signals to the frequency domain. The EEG data were analyzed over 64 electrode positions in the alpha (8–12 Hz) frequency band, as the alpha band has been found to be closely associated with mood [107, 108].

Data with absolute alpha power were normalized by log-transformation. The normalized absolute alpha values were averaged to generate one global and three regional mean values corresponding to the anterior (Fp1, Fp2, AF3, AF4, F1, F2, F3, F4, F5, F6, F7, F8, FPz, and Fz), centrotemporal (FC1, FC2, FC3, FC4, FC5, FC6, FT7, FT8, C1, C2, C3, C4, C5, C6, T7, T8, CP1, CP2, CP3, CP4, CP5, CP6, TP7, TP8, FCz, Cz, and CPz), and posterior regions (P1, P2, P3, P4, P5, P6, P7, P8, PO3, PO4, PO5, PO6, PO7, PO8, O1, O2, Pz, POz, and Oz). The source of the absolute alpha power band computed from the measurements of scalp electrical potential was further analyzed using LORETA [109, 110] and expressed in terms of three-dimensional cortical current density, using Talairach Brain Atlas coordinates. To ascertain whether the scalp EEG sources of the alpha band differed between the experimental and placebo groups, within-group comparison was conducted between the baseline and poststimulation measurements using paired-sample *t*-tests of 2394 voxels with subject-wise normalization [111–114].

With regard to alpha-power asymmetry, the frontal asymmetry index was used, as in previous studies literature [89–99], to measure changes in asymmetric activation at the mid-frontal pair of electrode sites (F3 and F4) between the two groups (*Group*: placebo versus experimental) before and after stimulation (*Time*: baseline versus poststimulation). Positive emotion is associated with increased frontal alpha asymmetry. Each score was computed by subtracting the left-sided log-transformed alpha power value (F3) from the value for the right side (F4). A positive score due to a relatively higher alpha power value in the right frontal region was taken to indicate that left-sided brain activity exceeded right-sided brain activity, suggesting mood enhancement, whereas a negative score indicated the opposite brain-activity pattern, suggesting a decline in mood.

## 3. Results

### 3.1. Changes in Absolute Alpha Power

Table 1 shows the means and standard deviations of the log-transformed absolute alpha power values at the baseline and after stimulation for the placebo and experimental groups (*μ*V^2^). The baseline measurements revealed no significant differences in absolute alpha power in the anterior, centrotemporal, and posterior regions between the placebo and experimental groups (*P* > 0.05). However, the experimental group demonstrated a global decrease in absolute alpha power from the baseline after stimulation, *t*(19) = 2.192, *P* = 0.041. Specifically, the reduction was significant in the anterior (*t*(19) = 2.670, *P* = 0.015) and centrotemporal (*t*(19) = 2.179, *P* = 0.042) regions but not in the posterior region (*t*(19) = 1.841, *P* = 0.081). In contrast, no significant change in absolute alpha power from the baseline measurement was found in the placebo group after sham stimulation.

### 3.2. Source Analysis of Absolute Alpha Power

**Figure 2:**
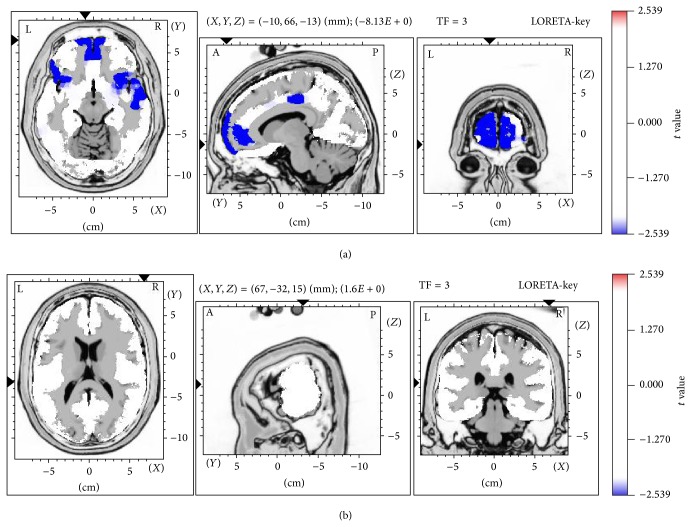
Reduced absolute alpha power in (a) the experimental group after stimulation as compared to the baseline but not (b) the placebo group. After stimulation at Shenmen acupoint, significant reduction occurred bilaterally in the superior frontal gyrus (BA 11, left: *X* = −10, *Y* = 66, *Z* = −13; BA 10, right: *X* = 9, *Y* = 66, *Z* = −10), medial frontal gyrus (BA 10, left: *X* = −8, *Y* = 66, *Z* = 4; right: *X* = 11, *Y* = 66, *Z* = −4), and inferior frontal gyrus (BA 47, left: *X* = −42, *Y* = 23, *Z* = −13; right: *X* = 35, *Y* = 18, *Z* = −13), as well as in the right superior temporal gyrus (BA 38, *X* = 50, *Y* = 16, *Z* = −13), right middle temporal gyrus (BA 21, *X* = 60, *Y* = 2, *Z* = −13), anterior cingulate (BA 32, *X* = −10, *Y* = 45, *Z* = −6), and cingulate gyrus (BA 24, *X* = −10, *Y* = −20, *Z* = 42). The most pronounced decrease was found in the left superior frontal gyrus (BA 11, *X* = −10, *Y* = 66, *Z* = −13). Blue color indicates the locations of significantly reduced absolute alpha power in the experimental group.

The LORETA voxel-by-voxel paired *t*-test with subject-wise normalization was used to examine the log-transformed alpha power separately for the experimental and placebo groups. As repeated tests were performed, the Bonferroni adjustment was used to set the alpha level to 0.025, with a corresponding significant *t*-value of 2.539 (df = 19). In the experimental group, LORETA analysis revealed a significant and consistent decline in absolute alpha power in both the frontal and centrotemporal regions after microcurrent stimulation at the Shenmen acupoint, compared with the equivalent baseline measurements (Figure 2(a)). The reduction was bilaterally in the superior frontal gyrus (BA 11, left: *X* = −10, *Y* = 66, and *Z* = −13; BA 10, right: *X* = 9, *Y* = 66, and *Z* = −10), medial frontal gyrus (BA 10, left: *X* = −8, *Y* = 66, and *Z* = 4; right: *X* = 11, *Y* = 66, and *Z* = −4), and inferior frontal gyrus (BA 47, left: *X* = −42, *Y* = 23, and *Z* = −13; right: *X* = 35, *Y* = 18, and *Z* = −13). Other brain regions included the right superior (BA 38, *X* = 50, *Y* = 16, and *Z* = −13) and middle temporal gyrus (BA 21, *X* = 60, *Y* = 2, and *Z* = −13), anterior cingulate (BA 32, *X* = −10, *Y* = 45, and *Z* = −6), and cingulate gyrus (BA 24, *X* = −10, *Y* = −20, and *Z* = 42).

### 3.3. Change in Frontal Alpha Asymmetry

**Figure 3:**
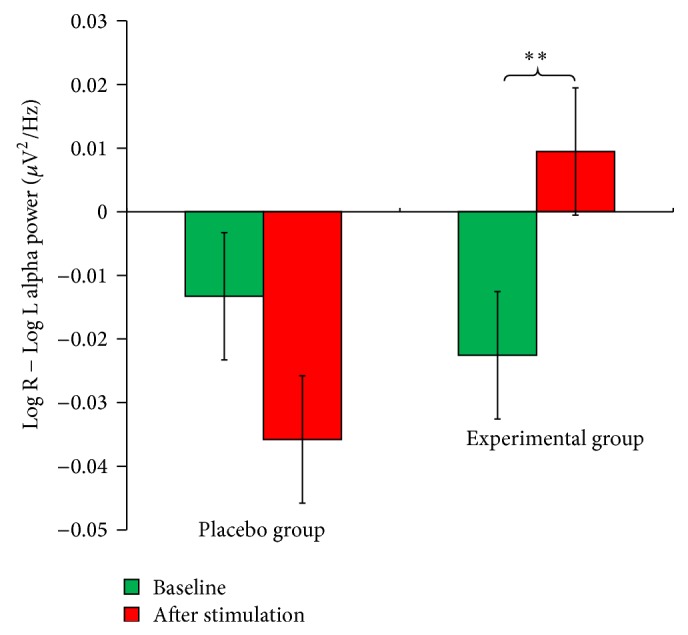
Frontal alpha asymmetry (F3-F4) at baseline and after stimulation for the placebo and experimental groups. A significant increase in the frontal alpha asymmetry score from the baseline to poststimulation was found in the experimental group, suggesting greater left-sided frontal brain activation associated with positive mood, after stimulation at the Shenmen acupoint (^**^ *P* < 0.01).

To investigate the changes in frontal (F3 and F4) alpha asymmetry, repeated-measures analysis of variance was conducted. The results revealed a significant* Time* ×* Group* interaction effect: *F*(1,38) = 11.253, *P* = 0.002. The results of subsequent paired-sample *t*-tests demonstrated a significant increase in the frontal alpha asymmetry score from the baseline to poststimulation in the experimental group, *t*(19) = −3.531, *P* = 0.002. However, this effect was not observed in the placebo group, *t*(19) = 1.668, *P* = 0.112 (Figure 3). An independent *t*-test revealed that there was no significant difference between the baseline measurements for the two groups, *t*(38) = 0.354, *P* = 0.725. Therefore, the change in frontal alpha asymmetry was not due to baseline differences, and the results suggest that the experimental group experienced greater left-sided frontal brain activation, associated with positive mood, after stimulation at the Shenmen acupoint.

## 4. Discussion

Microcurrent stimulation has been used as a novel alternative treatment for decades [1]. However, its mechanism of action remains unclear. The aim of this study was to investigate the electrophysiological effects associated with microcurrent stimulation at the Shenmen acupoint (HT7), using quantitative EEG measurement. Two objective electrophysiological indices, namely, absolute alpha power and frontal alpha asymmetry, were used to measure the EEG activities associated with arousal or sleepiness and mood.

The results of previous studies have suggested that insomnia is due to hyperarousal in the brain [84] and that patients with insomnia exhibit EEG brain activity that clearly distinguishes them from people without insomnia. Alpha activity, as a concomitant index of wakefulness and alertness, positively correlates with level of hyperarousal [86] and negatively correlates with subjective sleepiness [88]. Due to prolonged elevated alpha activity in the brain, patients with insomnia have difficulty falling asleep and tend to remain awake for extended periods. The results of this study indicated that the participants in the experimental group experienced a reduction in absolute alpha power after microcurrent stimulation at the Shenmen acupoint for 10 minutes. The reduction was particularly significant in the anterior and centrotemporal brain regions. However, this effect was not observed in the placebo group, whose members received sham stimulation. Therefore, the results suggest that microcurrent stimulation at the Shenmen acupoint affects EEG alpha activity, helping to reduce arousal level and thus activity. These positive findings offer initial electrophysiological evidence to explain the improvement in sleep observed in clinical populations and support the claim made by TCM practitioners that the meridian system is a pathway with a connection to the brain.

Researchers using positron emission tomography have found that compared with normal subjects, patients with insomnia show a smaller decline in relative metabolism during the transition from waking to non-REM sleep states in the ascending reticular activating system and the anterior cingulate and the medial prefrontal cortices [85]. Their functional-neuroimaging study provides objective evidence of hyperarousal in patients with insomnia and identifies brain regions that may assist in facilitating sleepiness in such patients. The results of EEG-based LORETA source analysis in the present study indicated that a reduction in absolute alpha power was found in these brain regions, including the bilateral medial prefrontal cortex and the anterior cingulate, after stimulation at the Shenmen acupoint. These encouraging findings shed some light on the electrophysiological effects localized at particular brain regions after stimulation at the Shenmen acupoint. However, as the study sampled normal participants without insomnia, further investigation of patients with insomnia will be necessary to determine whether stimulation at the Shenmen acupoint can really help to lower EEG arousal levels in the brain regions identified as elevated in patients with insomnia. Apart from changing EEG arousal activity, microcurrent stimulation at this acupoint was also found to lead to enhanced mood, as measured by frontal alpha asymmetry. This change was significant in the experimental but not the placebo group. These results are consistent with the explanation provided by TCM of Shenmen's therapeutic effects. TCM practitioners regard Shenmen as the gate to the spirit and claim that stimulation at the Shenmen acupoint can restore spiritual harmony. Recent studies on patients with insomnia after stroke reveal that acupuncture on Shenmen is able to lower the level of sympathetic activity, resulting in a significant decrease in heart rate variability [81, 82]. The possible mechanism seems to be related to specific afferent nerve signals sending to the central nervous system to lower the sympathetic activity [115]. In addition, an increase the serum level of serotonin in depressed patients with insomnia is shown after stimulation on Shenmen for four weeks with a period of 15 minutes twice a week. Though the underlying mechanism for the increase of serotonin requires further extensive investigation, the enhanced mood of the participants who participated in this study thus provides further empirical electrophysiological data in support of TCM observations related to Shenmen in reducing sleep disturbance [64, 77, 78] and improving mood [77, 78] over many years.

Although this randomized controlled study provides some initial insights into the electrophysiological effects of microcurrent stimulation at Shenmen, that is, its potential to lower arousal level, thereby facilitating sleepiness, and to enhance positive mood, the study has certain limitations that should be addressed. In this study, only alpha activity was used as measures for arousal level and emotional response. Apart from alpha power, theta and beta power are typically involved as an index of arousal as well [88, 116–118]. Specifically, significantly lower theta power and higher beta are found in patients with insomnia and positively correlated with their hyperarousal level [118]. It has been already proposed that increase in beta power may reflect the activity of brain structures involving in attentive behavior, leading to arousal [117, 119, 120]. Thus, in addition to using alpha power, future studies might be necessary to compare nonalpha bands, such as theta and beta bands, before and after stimulation. Second, the participants were blind to the group assignment, and changes in EEG activity were recorded, but they did not provide information on their subjective sleepiness or hyperarousal and mood. Given that sleepiness and mood questionnaires were not given to the participants in the experiment for reporting their sleepiness and mood states before and after stimulation, the correlation between EEG measures associated with arousal/mood and individuals' subjective emotional state is still unclear. Nevertheless, since the stimulation only lasted for 10 minutes, it is conceivable that change in subjective sleepiness and mood states may not be obviously noticed by the participants in the experiment. In future, longer period of stimulation, say once or twice per week for over four weeks, may be considered to measure if there is any change in their sleep pattern and mood state. Furthermore, insomnia is frequently associated with mood problems such as depression and anxiety [121, 122] and thus influences cognitive function, such as reducing attention [123]. Further investigation is necessary to determine whether stimulation at Shenmen has any effect on cognitive function. Future researchers should thus consider conducting clinical trials with patients with insomnia and mood problems by comparing EEG activity across different frequency bands (theta, alpha, and beta bands), subjective psychological states by self-administered questionnaires, and cognitive function before and after stimulation over several sessions and exploring the correlation between the objective and subjective measures.

## Figures and Tables

**Table 1:** Mean and standard deviation for log-transformed absolute alpha power during baseline and after stimulation for the placebo and experimental groups (*μ*V^2^).

Region	Placebo group (*N* = 20)	Experimental group (*N* = 20)
Anterior		
Baseline	0.696 (0.193)	0.725 (0.166)
After stimulation	0.675 (0.220)	0.657 (0.178)^*^
Centrotemporal		
Baseline	0.357 (0.254)	0.375 (0.210)
After stimulation	0.329 (0.245)	0.308 (0.177)^*^
Posterior		
Baseline	0.736 (0.275)	0.862 (0.319)
After stimulation	0.712 (0.256)	0.742 (0.247)
Global		
Baseline	0.556 (0.232)	0.611 (0.221)
After stimulation	0.531 (0.235)	0.527 (0.184)^*^

Values in the table are means with SD in parentheses.

Within-group comparison between baseline and after stimulation ^*^ *P* < 0.05.
